# AGE-induced neuronal cell death is enhanced in G2019S LRRK2 mutation with increased RAGE expression

**DOI:** 10.1186/s40035-018-0106-z

**Published:** 2018-01-23

**Authors:** Hyun Jin Cho, Chengsong Xie, Huaibin Cai

**Affiliations:** 1 Department of Biochemistry and Biomedical Sciences, Seoul National University, College of Medicine, 28 Yungun-dong, Jongro-gu, Seoul, 110-799 South Korea; 2 Transgenics Section, Laboratory of Neurogenetics, National Institute on Aging, National Institutes of Health, Building 35, Room 1A112, MSC 3707, 35 Convent Drive, Bethesda, MD 20892–3707 USA

**Keywords:** Parkinson’s disease, LRRK2, G2019S, AGE, RAGE, Neuronal death

## Abstract

**Background:**

Leucine-rich repeat kinase 2 (LRRK2) mutations represent the most common genetic cause of sporadic and familial Parkinson’s disease (PD). Especially, LRRK2 G2019S missense mutation has been identified as the most prevalent genetic cause in the late-onset PD. Advanced glycation end products (AGEs) are produced in high amounts in diabetes and diverse aging-related disorders, such as cardiovascular disease, renal disease, and neurological disease. AGEs trigger intracellular signaling pathway associated with oxidative stress and inflammation as well as cell death. RAGE, receptor of AGEs, is activated by interaction with AGEs and mediates AGE-induced cytotoxicity. Whether AGE and RAGE are involved in the pathogenesis of mutant LRRK2 is unknown.

**Methods:**

Using cell lines transfected with mutant LRRK2 as well as primary neuronal cultures derived from LRRK2 wild-type (WT) and G2019S transgenic mice, we compared the impact of AGE treatment on the survival of control and mutant cells by immunostaining. We also examined the levels of RAGE proteins in the brains of transgenic mice and PD patients by western blots.

**Results:**

We show that LRRK2 G2019S mutant-expressing neurons were more sensitive to AGE-induced cell death compared to controls. Furthermore, we found that the levels of RAGE proteins were upregulated in LRRK2 G2019S mutant cells.

**Conclusions:**

These data suggest that enhanced AGE-RAGE interaction contributes to LRRK2 G2019S mutation-mediated progressive neuronal loss in PD.

## Background

Parkinson’s disease (PD) is the second most common neurodegenerative disease with progressive loss of pigmented dopaminergic (DA) neurons in the substantia nigra pars compacta (SNpc) [1]. Although the majority of PD cases are sporadic, genetic studies of familial PD patients have identified mutations in more than 15 genes as causal factors for PD [2, 3]. Among PD-related causal genes, leucine-rich repeat kinase 2 (LRRK2) is the most common genetic cause of sporadic and familial PD, as well as the late-onset PD [4]. Up to now, more than 100 mutations of LRRK2 have been reported to be related to PD. Especially, the G2019S mutation has been identified as the most prevalent genetic cause of familial and sporadic PD [5]. Despite the relatively high prevalence, the penetrance of LRRK2 G2019S mutation is incomplete and age-dependent [5]. Therefore, it has been speculated that aging-related factors could also contribute to G2019S LRRK2-linked PD pathogenesis.

Advanced glycation end products (AGEs), raised from the reaction of sugars with certain amino acids or fats, are formed in high amounts in diabetes and also in the physiological organism during aging [6–9]. They have been implicated in numerous diabetes- and aging-related disorders such as cardiovascular disease, renal disease, and neurological disease by inducing oxidative stress, inflammation, and cell death [10–13]. Receptor of AGEs (RAGE), which belongs to the trans-membranous receptor of the immunoglobulin superfamily, is activated by several ligands including amyloid beta oligomers, calcium-binding proteins (S100 Calgranulins), and high-mobility group box-1 protein (HMGB1) as well as AGEs [14, 15]. Moreover, RAGE reveals high expression levels in neurons in neurodegenerative disorders, such as PD and Alzheimer’s disease (AD) [16]. In pathological environments, like PD and AD, RAGE expression is often upregulated with increased amounts of its ligands as well. Engagement of RAGE by AGE has shown to generate reactive oxygen species (ROS) via RAGE-mediated intracellular signaling and to accelerate pro-inflammatory events in cells.

Based on these earlier studies, we hypothesized that AGE-RAGE interaction might contribute to progressive neuronal cell death in G2019S LRRK2 mutation-expressing cells. In this study, we used LRRK2 G2019S overexpressing mouse model for in vitro neuronal culture experiments and demonstrated that LRRK2 G2019S mutant-expressing neurons were more sensitive to AGE-induced toxicity compared to the controls. In addition, RAGE levels were upregulated in LRRK G2019S mutant cells, suggesting enhanced AGE-RAGE activation is involved in LRRK2 G2019S mutation-related progressive neuronal loss.

## Methods

### Cell line and mouse primary neuron culture

HEK293 cells were culture in Dulbecco’s modified Eagle’s medium (DMEM, Gibco) containing a high glucose concentration supplemented with 10% fetal bovine serum (FBS) and penicillin/streptomycin. For the transfection, Fugene HD (Roche) was used. Primary neurons from cortex and striatum were prepared from newborn pups (postnatal day 0) [17]. Briefly, cells were dissociated by papain (Worthington Biochemical Corp) solution and then placed in poly-D-lysine (BD Bioscience) plate in Basal Eagle Medium (Sigma-Aldrich) supplemented with 1 mM L-glutamine, B27, N2, and penicillin/streptomycin (Invitrogen). To prevent glial cell growth, arabinosylcytosine (Sigma-Aldrich) was used. The medium was changed every 2 days.

### AGE preparation

To prepare AGE, BSA (50 mg/mL) was incubated with 0.5 M glucose in 0.2 M sodium phosphate buffer pH 7.4 for 10 weeks at 37 °C. The control sample of albumin was also incubated under same conditions but without glucose. All incubations were performed under sterile environments.

### Cell death assays

To determine the number of viable HEK293 cells, trypan blue dye exclusion assay was performed. It is based on the principle that live cells possess intact cell membrane that excludes dye whereas dead cells do not. A 1:1 dilution of HEK293 cell suspension was mixed with 0.4% Trypan Blue solution (Bio-Rad) at room temperature for 1-2 min. Viable cells showed clear cytoplasm, whereas nonviable cells were blue. Cell mixtures were injected into the hemocytometer chamber, and counted under the microscope. To assess the survival of primary cultured neurons, we seeded the same number of neurons in each culture and randomly captured the images for about 100 MAP2-stained neurons in vehicle and AGR-treated groups. We counted the number of survived neurons based on the appearance of normal dendritic morphology, mainly the continuous and elaborated dendritic trees.

### Generation of LRRK2 inducible transgenic mice

The cDNA fragments encoding full-length human G2019S LRRK2 mutant was inserted into a tetracycline operator-regulated gene expression vector, pPrP-tetP. To facilitate protein identification, C-termini of human G2019S LRRK2 protein were tagged by hemagglutinin (HA) epitope. The F1 transgenic mice were crossed with CaMKII-tTA mice to achieve high expression of LRRK2 G2019S in the forebrain [18]. The mice were fed regular diet ad libitum and housed in a 12 h light/dark cycle. All mouse work followed the guidelines approved by the Institutional Animal Care and Use Committees of the National Institute of Child Health and Human Development.

### Immunofluorescence staining

For immunocytochemistry, cultured neurons were fixed with 4% paraformaldehyde (PFA) and permeabilized with 0.1% Triton X-100 in PBS. After blocking nonspecific staining using 10% goat serum (Sigma-Aldrich) for 1 h, cells were incubated with primary antibodies overnight. For immunohistochemistry, mice were perfused via cardiac infusion with 4% PFA in cold PBS. To prepare frozen sections, brain was removed and submerged in 30% sucrose for 24 h and then sectioned at 40 μm thickness using cryostat (Leica CM1950). Primary antibodies used included MAP2 (SantaCruz), GFAP (Sigma-Aldrich), and NeuN (Millipore). To detect RAGE protein, primary antibodies from SantaCruz and R&D were used. Anti-LRRK2 polyclonal antibodies (4EC9E and 4C84E) were kindly provided from Dr. Jean-Marc Taymans. Alexa 488 or 568-conjugated secondary antibody was incubated to visualize the staining. Fluorescent images were captured by a Zeiss confocal microscope (LSM 510 META).

### Preparation of protein extracts and western blot

Mouse brain tissue was homogenized with 10 volumes of sucrose buffer (0.32 M sucrose, 1 mM MgCl_2_, 1 mM NaHCO_3_, and 0.5 mM CaCl_2_) containing protease and phosphatase inhibitor cocktail (Roche). To obtain crude membrane fraction, homogenized brain samples were centrifuged at 1000 × g for 10 min to remove nuclear fraction and then supernatants were centrifuged at 20,000 × g for 20 min. The pellet fraction was dissolved in RIPA buffer by sonication and concentrations were measured by BCA assay. Proteins were analyzed by 4-12% NuPage BisTris-polyacrylamide gel electrophoresis (Invitrogen) in MOPS running buffer (Invitrogene) and transferred to polyvinylidene difluoride (PVDF) membranes. The signals were visualized by enhanced chemiluminescence development (Pierce) and quantified using Scion Image System (Frederick, MD).

### Human brain tissues

Striatal tissues were obtained from the brain bank of Johns Hopkins University School of Medicine. Subjects or their legal representatives signed informed consents approved by the Johns Hopkins Institutional Review Boards. The diagnosis of PD was made based on the clinicopathological criteria including characteristic clinical features and on the presence of Lewy bodies within the pigmented neurons lost in the substantia nigra. The average age at death for the PD subjects included in this study was 80 + 6.9 years old, while for the NPC subjects it was 85 + 6.6 years old.

### Statistical analysis

Statistical analysis was performed with GraphPad Prism 5 (GraphPad Software). Statistical significances were obtained by comparing means of different case using t test or ANOVA followed by Tukey’s honestly significant difference test. Error bars indicate SD. ^*^P < 0.05; ^**^P < 0.01; ^***^P < 0.001.

## Results

### Characterization of AGE in cultured primary neurons

**Fig. 1:**
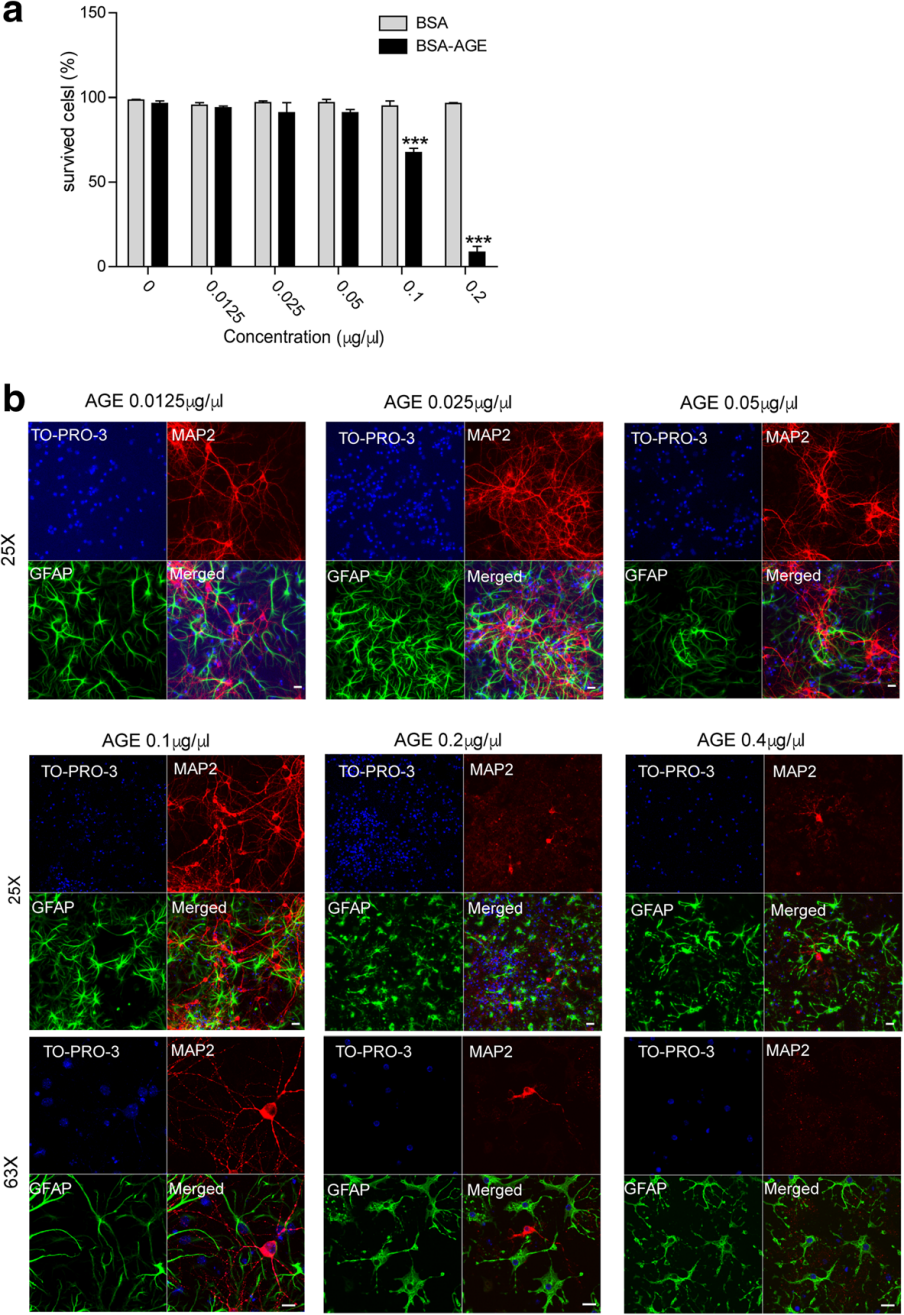
Characterization of prepared AGEs. **a**. HEK293 cells were incubated with BSA or BSA-AGE in a dose-dependent manner (0, 0.125, 0.25, 0.5, 1, 2 μg/μl). After 24 h, cells were detached from the plates using trypsin. Harvested cells were counted by trypan blue staining. Survived cells were presented in the bar graph. Data are presented as mean ± SD for three independent experiments. ^***^P < 0.001. **b**. Representative images show AGE-treated neurons. Neurons were incubated with AGE in a dose-dependent manner (0, 0.125, 0.25, 0.5, 1, 2 μg/μl). After 24 h, primary striatal neurons (15 DIV) were immunostained with MAP2 (red) and GFAP (green) antibodies. Scale bar, 20 μm

To test the toxic effects of AGEs generated for the cell viability assay, we first treated HEK293 cells with increasing dose of AGEs (Fig 1a). After treated with AGEs for 24 h, more HEK293 cells were lost at higher AGE dosages, whereas cells incubated with BSA showed no obvious cell death (Fig. 1a). Since LRRK2 is more abundant in the striatum [19], we cultured neurons from striatum of postnatal day 0 (P0) pups for AGE treatment. After 15 days in vitro culture (15 DIV), the cultured striatal neurons were incubated with AGEs at the concentrations of 0.0125, 0.025, 0.05, 0.1, and 0.2 μg/μl for 24 h and the cells were immuno-stained with anti-MAP2 and anti-GFAP antibodies to count the surviving neurons and astrocytes, respectively (Fig 1b). Cultured striatal neurons showed a number of neurites and healthy morphologies at the 0.0125 to 0.05 μg/μl AGE treatment. At the 0.1 μg/μl AGE-treated cases, we observed fragmented neurites but not neuronal loss. By contrast, almost all MAP2-positive neurons were dead after incubated with 0.2 and 0.4 μg/μl AGE for 24 h. On the other hand, GFAP-positive astrocytes were less sensitive to AGEs on the same condition. Based on these findings, in the later experiments, we treated cultured striatal neurons with AGE at the concentrations of 0.1~ 0.15 μg/μl for 24 h.

### LRRK2 G2019S-expressing neurons are more sensitive against AGE

**Fig. 2:**
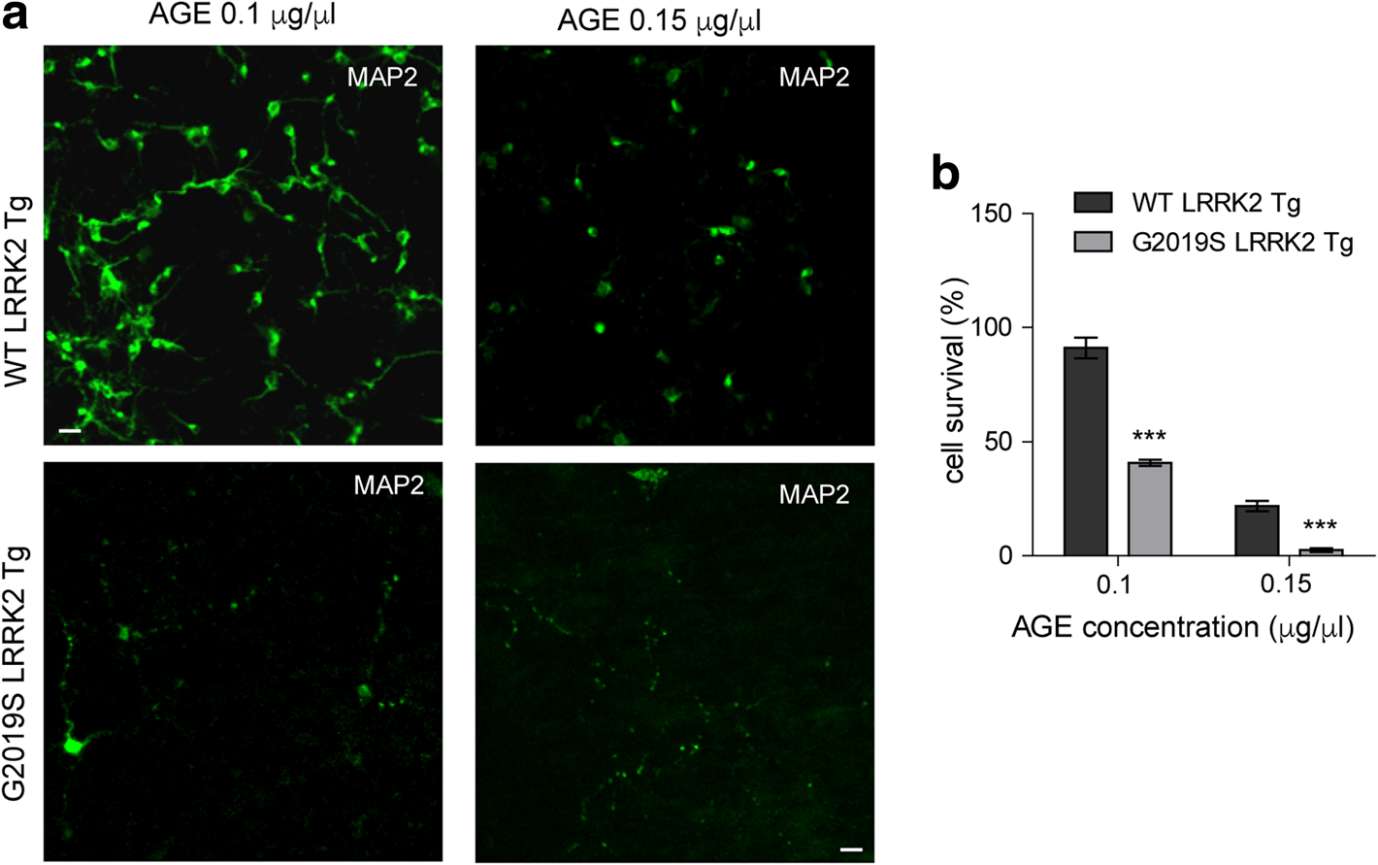
G2019S LRRK2 neurons show increased cell death against AGEs. **a**. Representative images show 0.1 μg/μl or 0.15 μg/μl AGE-treated neurons. Neuron cultures were derived from striatum of WT LRRK2 or G2019S LRRK2 transgenic pups. After 24 h, neurons (15 DIV) were immunostained with MAP2 (green) antibody. Scale bar, 40 μm. **b**. Bar graph shows quantification of survived cells in (**a**). Data are presented as mean ± SD for three independent experiments. ^***^P < 0.001

Next, we cultured the striatal neurons (DIV 15) from the striatum of P0 G2019S LRRK2 transgenic (Tg) and wild-type (WT) LRRK2 Tg pups to investigate whether these neurons exhibit different vulnerability to AGEs at 0.1 and 0.15 μg/μl after 24 h treatment (Fig. 2a). We found more than 60% loss of G2019S LRRK2-expressing neurons compared to the WT LRRK2 cultures after treated with 0.1 μg/μl AGEs for 24 h (Fig. 2a, b). The surviving G2019S LRRK2-expressing neurons exhibited severe neurite fragmentation and condensed cell body, while control neurons showed only slight neurite fragmentation (Fig. 2a). After treated with AGEs at 0.15 μg/μl for 24 h, more severe loss of G2019S LRRK2-expressing neurons as well as WT neurons was observed (Fig. 2a, b). These data indicate that G2019S LRRK2-expressing neurons are much more sensitive to AGE –induced toxicity compared to neurons expressing WT LRRK2.

### AGE-induced neuronal cell death is mediated by RAGE

**Fig. 3:**
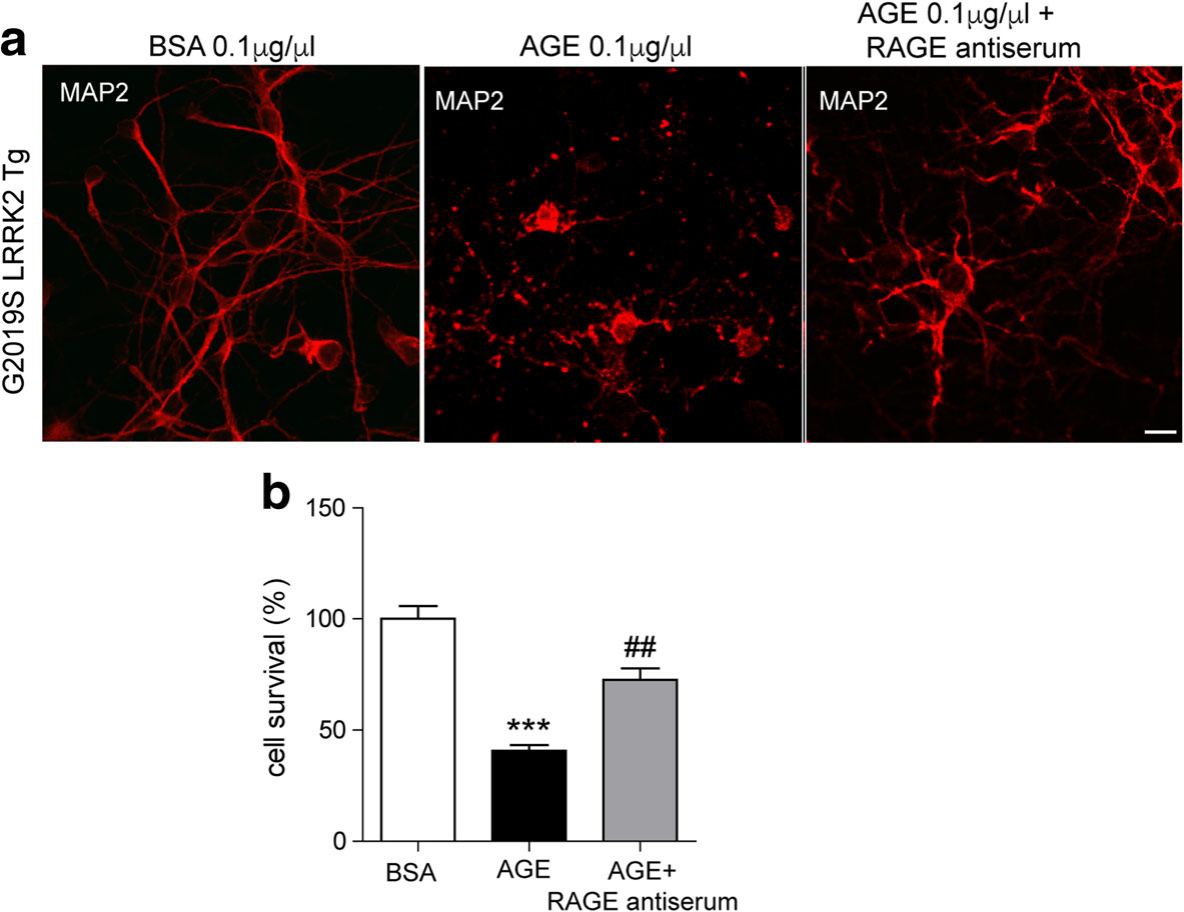
RAGE neutralizing antibody prevents AGE-induced neuronal death. **a**. Representative images show 0.1 μg/μl BSA or AGE-treated G2019S LRRK2 neurons. RAGE neutralizing antiserum pretreated for 30 min. After 24 h, neurons (15DIV) were immunostained with MAP2 (red) antibody. Scale bar, 20 μm. **b**. Bar graph shows quantification of survived cells in (**a**). Data are presented as mean ± SD for three independent experiments. ^***^P < 0.001 compared with BSA-treated cells. ^##^*P* < 0.01 compared with AGE-treated cells

We then investigated whether AGE-induced neuronal cell death in LRRK2 G2019S neurons is mediated by any specific intracellular signaling pathways. Receptor of AGE (RAGE) is reported to be expressed in aged brains. Therefore, we hypothesized that neuronal cell death triggered by AGE is mediated by RAGE. To test the role of RAGE, we neutralized RAGE function using anti-RAGE antiserum in LRRK2 G2019S neurons (Fig. 3a). As a result, severe cell death induced by 0.1 μg/μl AGE treatment was remarkably mitigated by pre-incubation of anti-RAGE antiserum (Fig. 3a, b). This event indicates that RAGE-related pathway participates AGE-induced neuronal cell death in LRRK2 G2019S mutation.

### Upregulation of RAGE protein levels in G2019S LRRK2 samples

**Fig. 4:**
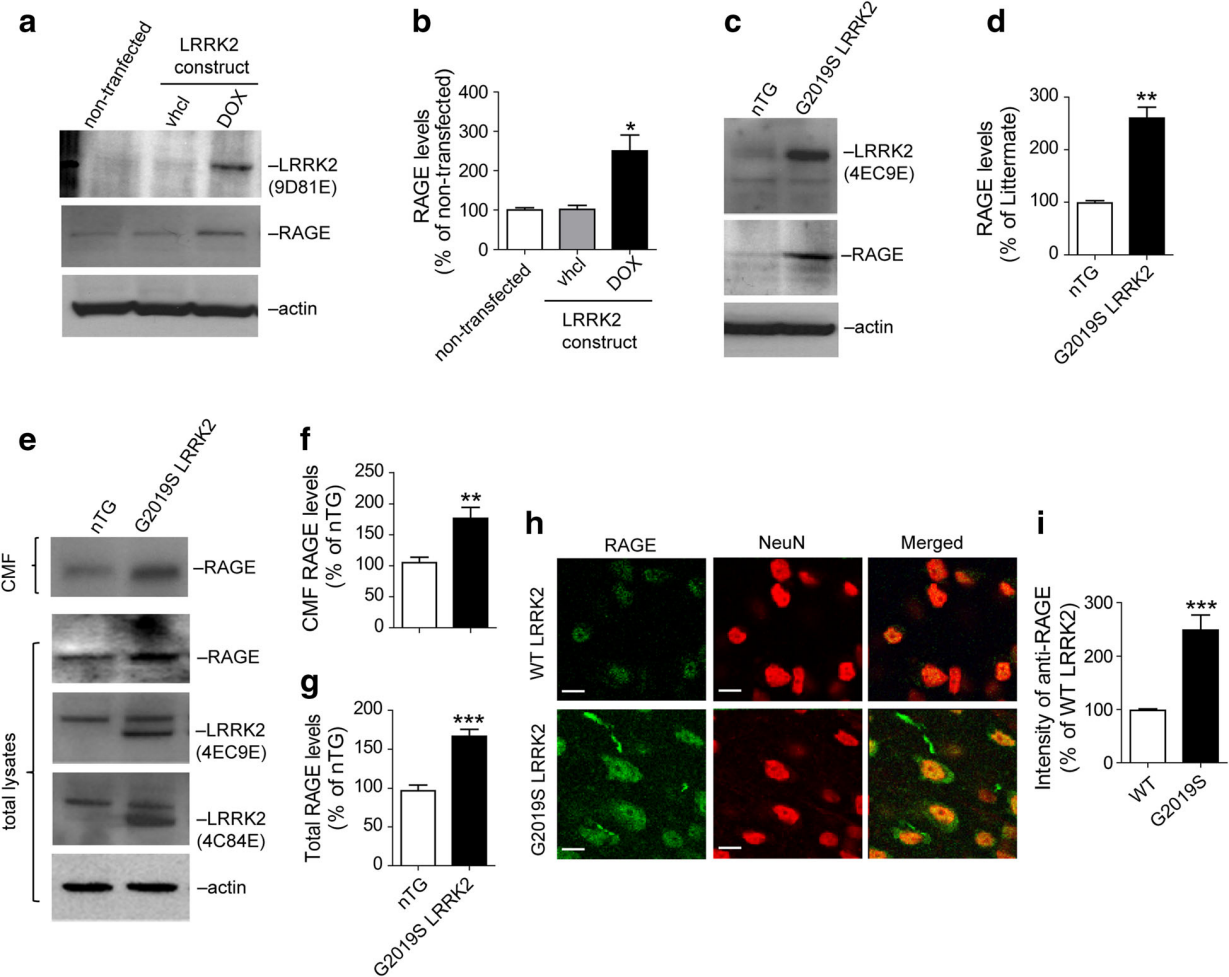
G2019S LRRK2 cells show up-regulation of RAGE protein levels. **a**. Western blot analysis of RAGE in doxycycline (DOX)-activated G2019S-LRRK2 expressing HEK293 cells. After 48 h transfection, cells were lysed and analyzed. **b**. Bar graph shows quantification of (**a**). Data are presented as mean ± SD for three independent experiments. ^*^P < 0.05. **c**. Western blot analysis of RAGE in cultured primary neurons (DIV 15) from striatum of nTG mice and G2019S LRRK2 transgenic mice. **d**. Bar graph shows quantification of (**c**). Data are presented as mean ± SD for three independent experiments. ^**^P < 0.01. **e**. Western blot analysis of RAGE in protein lysates from striatum of nTG and G2019S LRRK2 transgenic mice. Total lysates and crude membrane fraction (CMF) were analyzed. **f**. Bar graph shows quantification of RAGE levels from CMF lysates in (**e**). Data are presented as mean ± SD for three independent experiments. ^**^P < 0.01. **g**. Bar graph shows quantification of RAGE levels from total lysates in (**e**). Data are presented as mean ± SD for three independent experiments. ^***^P < 0.001. **h**. Representative images show RAGE in striatal neurons (NeuN-positive cells) from the brain sections of 3-month-old WT LRRK2 and G2019S LRRK2 transgenic mice. Neurons in the striatum region immunostained with NeuN (red) and RAGE antibodies (green). Scale bar, 20 μm **i**. Bar graph shows quantification of (**h**). Data are presented as mean ± SD for three independent experiments. ^***^P < 0.001

To test whether RAGE protein levels are regulated by G2019S LRRK2 expression, HEK293 cells were transfected with doxycycline (DOX)-activated G2019S LRRK2 expression constructs (Fig. 4a). We found enhanced RAGE protein levels in cells treated by DOX (Fig. 4a, b). Also, we observed increased levels of RAGE proteins in the G2019S LRRK2 expressing striatal neuron cultures compared to control neurons (Fig. 4c, d). Next, the striatal tissues from 3-month-old G2019S LRRK2 transgenic and littermate non-transgenic (nTG) mice were dissected and total proteins were extracted for analyses of RAGE expression. Since RAGE is a type I transmembrane protein, crude membrane proteins were also prepared to examine the RAGE protein levels. Significantly increased RAGE levels were presented in both total lysates and crude membrane extracts of the brains from G2019S LRRK2 mutant mice (Fig. 4e-g). Furthermore, immuno-staining with anti-RAGE antibody also confirmed the increase of RAGE levels in the cultured striatal neurons from G2019S LRRK2 P0 pups compared to the WT LRRK2 (Fig. 4h, i). These results provide strong evidence of upregulated RAGE protein expression in the G2019S LRRK2-expressing mouse brains.

### Increased level of RAGE protein in the brains of PD patients

**Fig. 5:**
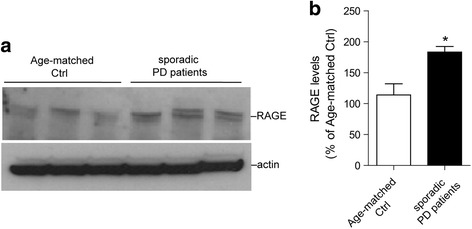
Increased RAGE level in brains of sporadic PD patients. **a**. Western blot analysis of RAGE in total lysates from striatum of PD patients (*n* = 3) and age-matched control (Ctrl) subjects. (*n* = 3). **b**. Bar graph shows quantification of RAGE levels in (**a**). Data are presented as mean ± SD for three independent experiments. ^*^P < 0.05

Finally, we examined the levels of RAGE proteins in the human brain samples from sporadic PD patients and age-matched control subjects. We tested total protein lysates extracted from striatal tissues. As expected, significantly increased RAGE expression in the striatum from sporadic PD patients was detected by the western blotting (Fig. 5). This upregulation of RAGE protein levels in the striatum of PD patients supports the involvement of AGE-RAGE pathway in PD pathogenesis.

## Discussion

Diverse mutations in LRRK2 underlie the most common genetic cause of familial and sporadic PD. Since the identification of PD-associated mutations in LRRK2, many studies have attempted to illustrate the potential pathogenic mechanisms of the neuronal cell death caused by mutant LRRK2, especially the G2019S mutation. Here, we demonstrated that AGE-induced toxicity in the striatal neurons was enhanced in the presence of G2019S LRRK2. Furthermore, we found that the level of RAGE, the critical mediator of AGE-induced cell death, was upregulated in the G2019S LRRK2-expressing neurons. This finding was confirmed in diverse experimental systems, including G2019S LRRK2-expressing cell lines, primary neurons, and mouse brain extracts, as well as postmortem PD striatal tissues. These results reveal a previously unknown pathogenic mechanism of LRRK2 G2019S mutation in AGE/RAGE-mediated cytotoxicity.

We detected elevated protein levels of RAGE but not mRNA levels (data not shown) in G2019S LRRK2, indicating the possibility that the upregulation of RAGE mRNA translation by G201S LRRK2, resulting from the pathologically enhanced kinase activity of G2019S mutation. Also, in support that increased RAGE protein levels are not the results from artificially over-expressed G2019S LRRK2, we checked the AGE-induced cell toxicity and RAGE levels in the wild-type LRRK2 overexpressing neurons. We found significant changes of RAGE levels between wild-type LRRK2 and G2019S LRRK2 overexpressing neurons, indicating that this event is not resulted from artificial transgene expression.

AGEs, the senescent protein derivatives are formed at accelerated rate under normal aging process, which elicit reactive oxygen species generation and inflammation, and subsequently alters diverse gene expressions, apoptotic neuronal cell death [20–22]. It has been reported that AGE-albumin, the most abundant AGE product, is synthesized and secreted from the cells and studied as a key inducer of host cell death in various neurodegenerative disorders by enhanced expression of RAGE [23]. Gomez et al. showed increased level of RAGE in the frontal cortex of sporadic PD brains [16]. In line with this early study, we found increased RAGE protein levels in the striatum of PD patients, as well as LRRK2 G2019S transgenic mice at 3 months of age.

## Conclusions

In summary, we demonstrate that AGE-RAGE cascades are involved in G2019S LRRK2-mediated pathogenesis in PD. Our study suggests that targeted pharmacological interventions using inhibitors or antagonist, such as RAGE neutralizing antiserum, or inhibitors against AGE-RAGE intracellular signaling may serve as promising therapeutic strategies to slow down the progression of PD, especially, PD patients having G2019 LRRK2 mutation.
